# Guidelines for laboratory rearing of insect evidence: the importance of air humidity for breeding of *Necrodes littoralis* (L.) (Coleoptera: Staphylinidae)

**DOI:** 10.1038/s41598-025-92196-1

**Published:** 2025-03-12

**Authors:** Anna Mądra-Bielewicz, Szymon Matuszewski

**Affiliations:** 1 Laboratory of Criminalistics, Adam Mickiewicz University, al. Niepodległości 53, Poznań, 61-714 Poland; 2 Center for Advanced Technologies, Adam Mickiewicz University, Uniwersytetu Poznańskiego 10, Poznań, 61-614 Poland

**Keywords:** Rearing protocols, Forensic entomology, Thermogenesis, Carrion beetles, Silphinae, Behavioural ecology, Animal behaviour, Animal physiology, Entomology

## Abstract

In forensic entomology, effective rearing of insect evidence is crucial as it increases the accuracy of post-mortem interval (PMI) estimation and facilitates species identifications of some evidence. However, virtually no single species has a forensically useful rearing protocol. This study investigates the importance of relative humidity on larval development and fitness of *Necrodes littoralis* (L.) (Staphylinidae), and proposes an all-inclusive rearing protocol for this forensically important beetle species. We hypothesized that high relative humidity enhances growth, shortens development and promotes thermogenesis. By manipulating relative humidity (50%, 70%, 90%) and the presence of adult beetles during the prelarval phase, we demonstrated that high relative humidity level significantly improved beetle fitness by increasing survival, beetle mass at eclosion and shortening development. Specifically, at 90% relative humidity, beetles showed a twofold increase in mass and a threefold increase in survival compared to 50%. Moreover, thermogenesis was higher at high relative humidity, further facilitating larval growth. These results highlight the key role of humidity for *N. littoralis* development. Finally, we proposed a comprehensive rearing protocol for *N. littoralis* to improve forensic investigations involving these beetles and to guide future developments of similar protocols for other insects of forensic importance.

**Supplementary Information:**

The online version contains supplementary material available at 10.1038/s41598-025-92196-1.

## Introduction

A protocol is a detailed set of rules and guidelines developed for proceeding in a specific situation. In forensic entomology, several recommendations have been published for collecting and preserving insect evidence^1,2^. They include mostly general instructions for higher taxa, rather than specific protocols for genera or species. In the case of insect rearing, the information is basic or scattered in different sources and we still lack high-standard protocols including all biotic and abiotic elements relevant for effective rearing. Certainly, some general information can be found in forensic entomology textbooks^3–5^ and articles^1,6,7^. However, there are almost no laboratory protocols that provide comprehensive information on how to rear a specific genus or species of forensically-important insects. Developing insect rearing protocols in forensic entomology is important for several reasons. First, the know-how in rearing is often crucial to identify immature life stages found at the death scene, since some of them can be easily identified to the species level after they reach the adult stage. Moreover, further rearing of preimaginal forms collected at a death scene allows for a more accurate estimation of their age and the minimum PMI^8^. Finally, understanding and maintaining optimal rearing conditions for a given species is necessary for experimental elaboration of insect developmental models useful in estimating PMI.

To develop a species-specific insect rearing protocol, basic knowledge on its biotic and abiotic elements is needed. The most important biotic elements are: diet (whether a life stage is predatory or necrophagous, what prey is optimal as food regarding predatory life stages etc.) and colony abundance (does it feed individually or in aggregations regarding necrophagous life stages, what size of aggregation is optimal under laboratory conditions etc.). The most important abiotic elements include: temperature, humidity and photoperiod. These parameters are relatively simple to control with the use of climatic chambers. Nevertheless, their optimal ranges can only be determined through time-consuming developmental studies.

Most of the laboratory studies on the development of forensically important beetles have only focused on the effect of temperature, with the relative air humidity held constant or uncontrolled (Table 1). We are aware of only three studies, in which the authors investigated the influence of relative air humidity on the development of forensically important beetles, all regarding larder beetles (Coleoptera: Dermestidae)^9–12^. Apart from beetles important for stored-products management e.g. species of *Necrobia* or *Dermestes*^13,14^, fly species important for biowaste recycling, e.g. *Hermetia illucens* (L.) or *Musca domestica* L.^15–18^ and biosurgery, e.g. *Lucilia sericata *(Meigen)^19,20^, the typical necrophagous species do not have high-standard rearing protocols.

**Table 1:** List of developmental studies on forensically important beetle species according to air temperature and humidity.

No.	Family	Species	Temperature	Humidity	Ref.
1.	Cleridae	*Necrobia ruficollis* F.	22, 25, 28, 31, 34	70	^47^
2.		*Necrobia rufipes* (Deg.)	22, 25, 28, 31	70–75	^48^
3.			22, 25, 28, 31, 34, 36	70	^49^
4.	Dermestidae	*Dermestes frischii* Kug.	19, 22, 25, 28, 31, 34	70	^50^
5.			23, 26	75	^51^
6.			15, 20, 25, 30, 35	60–80	^52^
7.			20, 22.5, 25, 27.5, 30, 32.5, 35, 37.5, 40	40, 50, 60, 70	^10^
8.		*Dermestes haemorrhoidalis* Küst.	12.5, 15, 20, 25, 30, 32.5	40, 50, 60, 65, 70, 80	^53^
9.		*Dermestes lardarius* L.	12.5, 15, 20, 25, 30, 32.5	40, 50, 60, 65, 70, 80	^11^
10.		*Dermestes maculatus* Deg.	19, 22, 25, 28, 31, 34	75	^54^
11.			20, 24, 28, 32	50–60	^55^
12.			15, 20, 25, 30, 35	60–80	^52^
13.			15, 20, 22, 24, 27, 30	55.4	^56^
14.			30	65	^57^
15.			15, 20, 25, 30, 35	60–80	^58^
16.			25, 28, 31, 34	0, 20, 50, 70, 100	^9^
17.		*Dermestes peruvianus* Cast.	12.5, 15, 20, 25, 30, 32.5	40, 50, 60, 65, 70, 80	^53^
18.		*Dermestes tessellatocollis* Motsch.	16, 19, 22, 25, 28, 31, 34	75	^59^
19.		*Dermestes undulatus* Brahm	23, 26	75	^51^
20.			15, 20, 25, 30, 35	60–80	^52^
21.	Histeridae	*Carcinops pumilio* (Erisch.)	17.5, 19.5, 25.5, 30, 32.5, 35	no data	^60^
22.		*Euspilotus azureus* (Sahl.)	10, 15, 20, 25, 30, 35	65	^61^
23.	Leiodidae	*Sciodrepoides watsoni* (Spence)	12, 15, 18, 21, 25, 28	no data	^62^
24.	Nitidulidae	*Nitidula rufipes* (L.)	16, 19, 22, 25, 28, 31, 34	70	^63^
25.		*Omosita colon* (L.)	16, 19, 22, 25, 28, 31, 34	70	^64^
26.	Staphylinidae	*Aleochara asiatica* Kraatz	17.5, 20, 22.5, 25, 27.5, 30	100	^65^
27.		*Aleochara nigra* Kraatz	17.5, 20, 22.5, 25, 27.5, 30	100	^65^
28.		*Creophilus maxillosus* (L.)	10, 12.5, 15, 17.5, 20, 22.5, 25, 27.5, 30, 32.5	60–70	^43^
29.			17.5, 20, 22.5, 25, 27.5, 30, 32.5	75	^66^
30.			16, 24, 32	50	^67^
31.		*Necrodes littoralis* (L.)	14, 15, 16, 17, 18, 19, 20, 22, 26, 30	no data	^30^
32.		*Necrophila brunnicollis* (Kraatz)	18, 20, 22, 29	no data	^68^
33.		*Oxelytrum discicolle* (Brullé)	15, 20, 28	70–80	^69^
34.		*Thanatophilus micans* (F.)	15, 22.5, 32.5	no data	^70^
35.			15, 17, 18, 19, 20, 22.5, 25, 28.4, 32.5, 35	no data	^71^
36.		*Thanatophilus mutilatus* (Cast.)	14, 15, 17.5, 19, 20, 22.5, 25, 27.5, 30	no data	^70^
37.		*Thanatophilus rugosus* (L.)	23, 26	32, 70	^12^
38.			12, 14, 16, 18, 20, 22	no data	^72^
39.		*Thanatophilus sinuatus* (F.)	14, 16, 18, 20, 23, 26, 29	50–60	^73^
40.			14, 16, 18, 20, 21, 24, 26	no data	^42^

*Necrodes littoralis* (L.) (Coleoptera: Staphylinidae: Silphinae) is a widely distributed Palaearctic species^21^. It is mainly present from spring to autumn (April–October), both in forest and open natural habitats^22–24^. It is highly useful in forensic entomology for several reasons: it frequently breeds on large vertebrate cadavers; it has been reported in many forensic case studies^22,25–28^, its appearance on a cadaver (i.e. the pre-appearance interval, PAI) is strongly dependent on the preceding ambient air temperature^29^ and it has forensically-useful temperature developmental models^30^. There is also a good understanding of its biology. The adults can quickly locate carrion, where they are able to effectively compete with blow flies over cadaver resources by preferentially killing late 2nd instar and early 3rd instar maggots^24^. They lay eggs in small batches, in the soil nearby the cadaver. Moreover, the adult *Necrodes littoralis* beetles can form a complex microenvironment on a cadaver (i.e. the feeding matrix), by spreading oral and anal exudates over carrion tissues. Formation of the matrix is crucial for the fitness of *N. littoralis* larvae, which further develop the matrix and modify its features. The larvae feed in larval aggregations, enhancing their fitness and accelerating carrion decomposition^31,32^. The heat produced in the matrix shortens larval development and can have other positive effects for larval fitness^33^. Formation of the matrix probably depends on various factors, including air humidity, with conditions of high humidity likely promoting its formation and enhancing thermogenesis. When larvae reach the postfeeding stage, they leave the carrion, bury themselves in the soil where they form pupal chambers and eventually pupate and eclose into adult beetles. In natural conditions, *N. littoralis* reveals a strong preference for carrion in humid habitats, for instance much higher abundances of these beetles were recorded on pig carcasses in alder forest (a humid marshy forest temporarily and partly flooded by groundwater) than in drier hornbeam-oak or pine-oak forests^24,34^.

As the majority of the elements of the rearing protocol are already known for *N. littoralis*, in the current study we investigated the importance of relative air humidity for its larval development and fitness. We hypothesized that high ambient air humidity improves offspring fitness, shortens larval development and enhances thermogenesis within the feeding matrix. Finally, using the current data we were able to develop a comprehensive laboratory-rearing protocol for *N. littoralis*, a species of high forensic importance.

## Results

### Beetle fitness

**Fig. 1:**
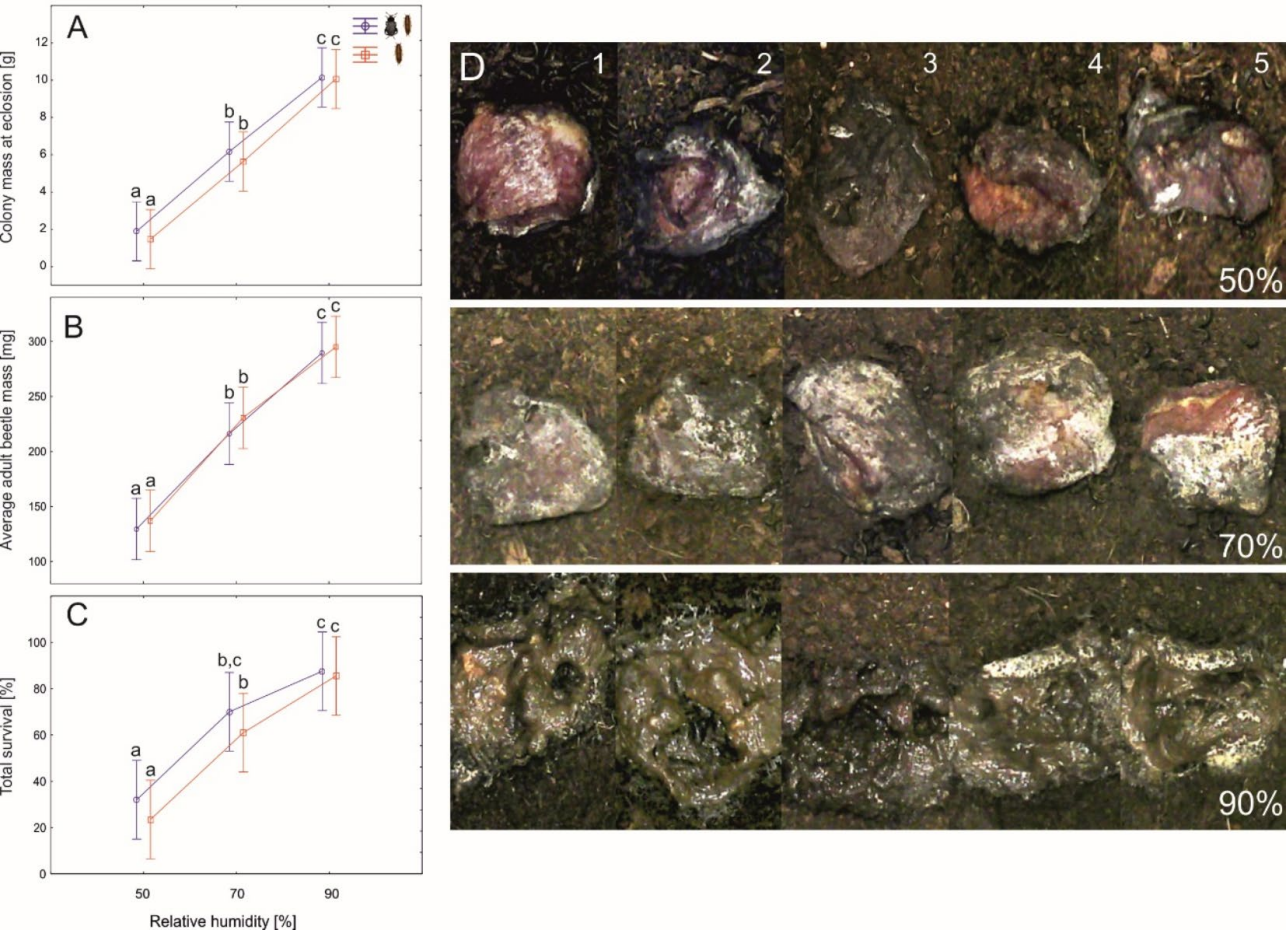
The differences in colony mass at eclosion (**A**), average adult beetle mass at eclosion (**B**) and total survival (**C**) between colonies of larval *N. littoralis* reared under various air humidity and with or without adult beetles in the prelarval phase. Symbols: means, whiskers – 95% confidence intervals; different letters denote significant differences in pairwise comparisons. (**D**) Decomposing pieces of meat in a setup with adult beetles present in the larval feeding phase under different humidity levels (lower right corners). Photographs were made between 216 and 240 h from the start of the experiment. Numbers 1–5 denote replicates.

Increasing relative air humidity (RH) significantly improved beetle fitness, expressed here as colony mass at eclosion (Fig. 1A; Table 2). This effect was a cumulation of two components: the average adult beetle mass at eclosion, which doubled at 90% RH compared to 50% RH (Fig. 1B) and the total survival, which tripled at 90% RH compared to 50% RH (Fig. 1C). Parental effects and interactions were insignificant in these analyses (Table 2). At the lowest RH the meat was desiccated and hardly accessible to the larvae (Fig. 1D). Most of the time the larvae were observed beneath the meat, sometimes they were able to form cavities in the underside of the meat, where they subsequently fed. The meat in the highest RH was the most decomposed, covered with a typical feeding matrix and easily accessible to the larvae (Fig. 1D).

**Table 2:** The results of the ANOVA test to assess the significance of ‘relative humidity’ and ‘parental effects’ on dependent variables.

Dependent variables	Independent variables				Interaction	
	Relative humidity		Parental effects			
	F	*p*	F	*p*	F	*p*
**Colony mass at eclosion**	60.00	< 0.001	0.31	0.59	0.05	0.95
**Average adult beetle mass**	69.60	< 0.001	0.69	0.41	0.06	0.93
**Total survival**	26.18	< 0.001	0.94	0.34	0.11	0.89
**Average total development time**	63.08	< 0.001	7.46	0.01	1.61	0.22
**Average larval developmental time**	61.92	< 0.001	6.67	0.02	2.01	0.16
**Average pupal development time**	9.11	0.001	0.06	0.81	0.97	0.39
**Average thermogenesis in the prelarval phase**	10.18	< 0.001	4.74	0.04	4.74	0.02
**Average thermogenesis in the larval phase**	31.18	< 0.001	5.39	< 0.001	1.00	0.38

**Fig. 2:**
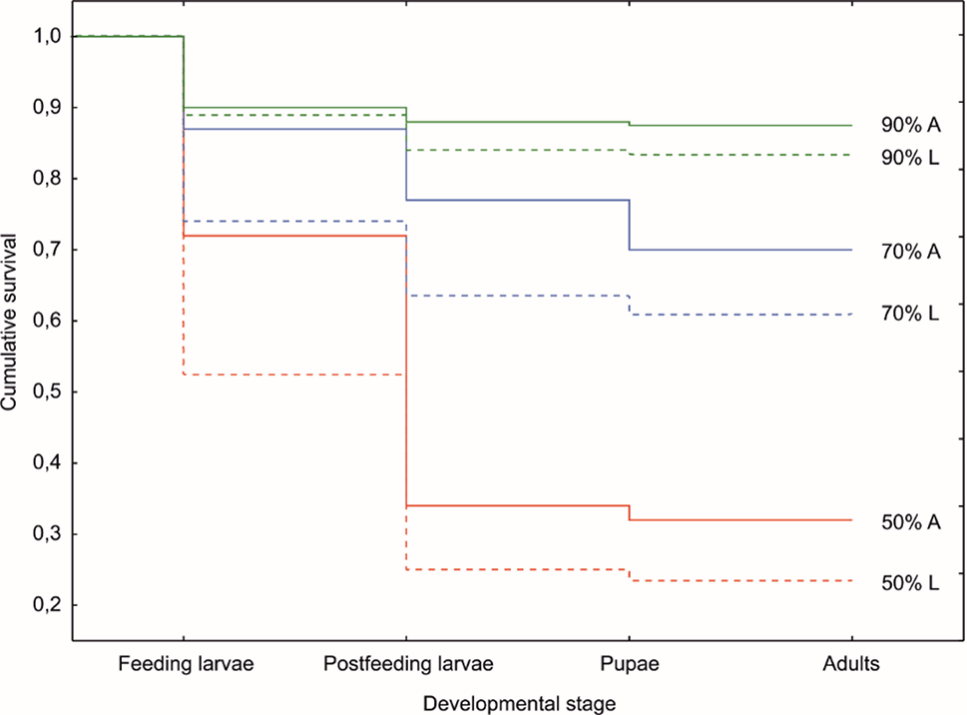
The survival of different developmental stages of *Necrodes littoralis* across three humidity levels. A - adults present in the prelarval phase; L - adults absent in the prelarval phase; (Kaplan - Meier analysis, *p* < 0.001).

Both feeding and postfeeding larvae were vulnerable to detrimental effects of low RH in the larval feeding phase (Fig. 2). Under conditions of low or medium RH the mortality of the feeding larvae (1st instar–3rd instar) was visibly higher in the setup without adult beetles in the prelarval phase. In the the highest relative air humidity these differences were much smaller (Fig. 2).

**Fig. 3:**
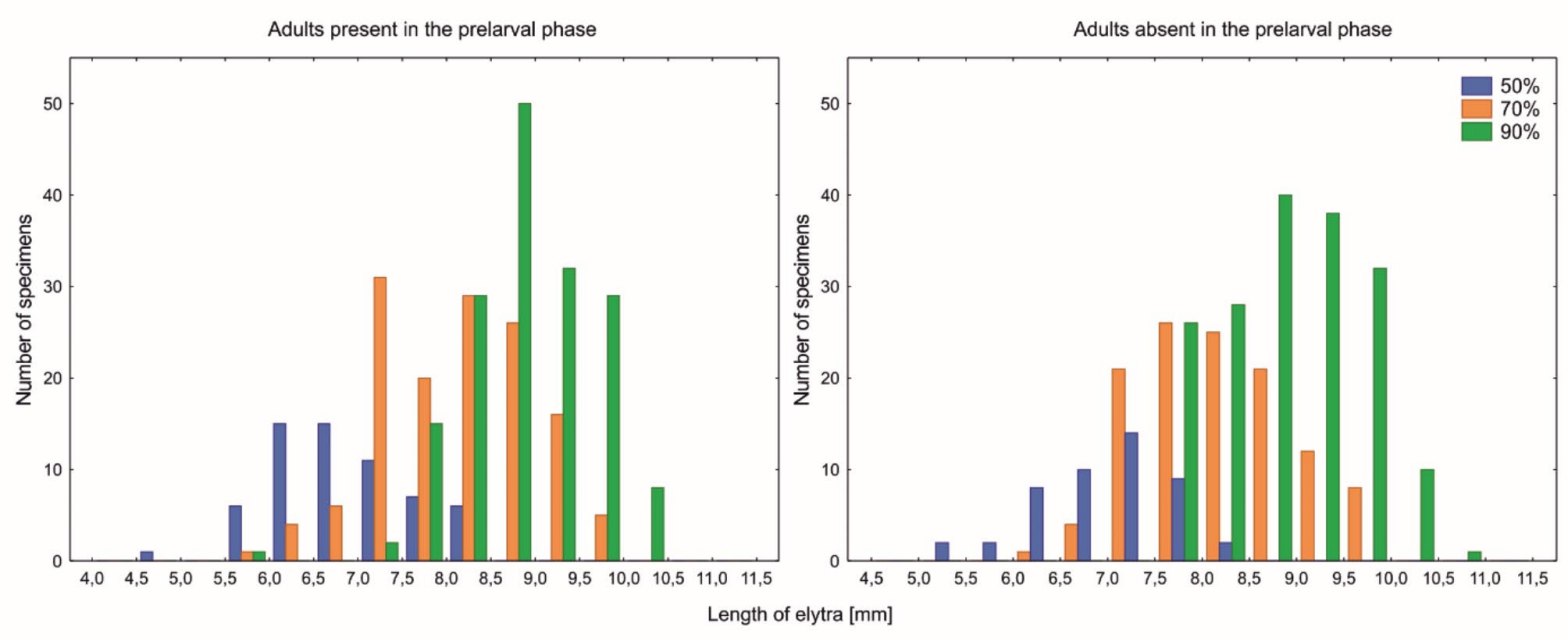
The distribution of sizes (i.e. length of elytra; averaged for left and right elytron) of *Necrodes littoralis* beetles at eclosion across colonies reared at three relative air humidity levels in setups with or without adult beetles in the prelarval phase.

In both setups (with and without adult beetles in the prelarval phase) the largest beetles at eclosion were observed at 90% RH (Fig. 3). The relative humidity had a significant influence on the size of beetles in both sexes (Supplementary Material: Table 1; Fig. 1).

### Development time

**Fig. 4:**
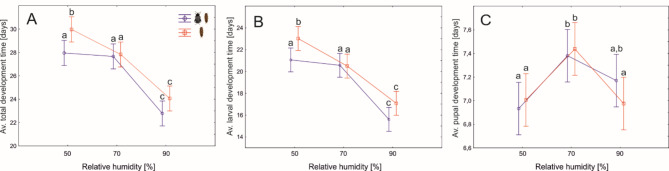
The differences in average (per colony) total (**A**), larval (**B**) and pupal (**C**) development times between colonies of *N. littoralis* reared under various air humidity and with or without adult beetles in the prelarval phase. Symbols: means, whiskers – 95% confidence intervals; different letters denote significant differences in pairwise comparisons.

The larval, pupal and total development times were significantly different according to the relative humidity (Table 2, Supplementary Material: Table 1; Fig. 1). The duration of larval and total development was inversely related to RH (Fig. 4A,B). Under high RH these development times were significantly shorter by about 5–6 days compared to low or medium RH. There was also a significant effect of the presence of adult beetles in the prelarval phase; however, the shortening of development in colonies with adult beetles was significant only under low RH (Fig. 4A,B; Table 2). The differences in pupal development times between RH levels were significant but very small (Fig. 4C; Table 2). The pupal development time in 70% RH was about 3–6% longer when compared to 50% RH and 6–7% longer when compared to 90% RH.

### Thermogenesis

**Fig. 5:**
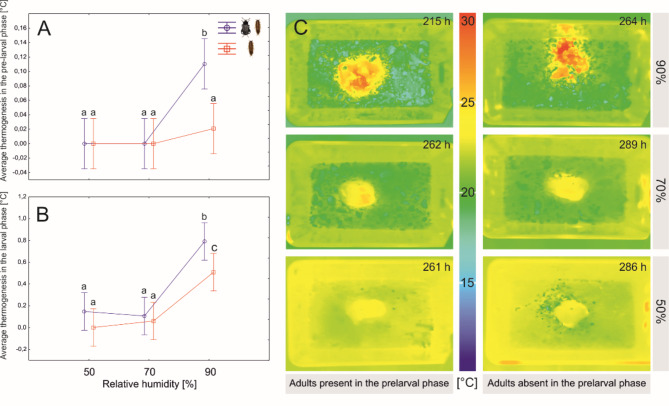
The differences in thermogenesis during the prelarval phase (**A**) and larval phase (**B**) between colonies of *N. littoralis* reared under various air humidity and with or without adult beetles in the prelarval phase. Symbols: means, whiskers – 95% confidence intervals; different letters denote significant differences in pairwise comparisons. (**C**) Thermal images taken in the day with the highest thermogenesis according to relative humidity and the presence of adult beetles in the prelarval phase.

The highest thermogenesis was observed in the colonies kept under the highest RH, both in the prelarval and larval phase, under low and medium humidity thermogenesis was virtually absent (Fig. 5A,B; Table 2). The presence of adult beetles had significant positive effect in this respect, which was revealed in both phases (Table 2). The highest thermogenesis was observed between 215 and 262 h after the establishment of colonies with adult beetles in the prelarval phase and between 264 and 289 h in the colonies without adult beetles in the prelarval phase (Fig. 5C).

No interaction between humidity and parental effects was significant, except for average thermogenesis in the prelarval phase, in case of which it simply indicates that under low and medium humidity there were no differences between parental effects groups, whereas under high humidity large difference was present (Table 2).

## Discussion

Our study shows the importance of high relative air humidity for the effective development of *Necrodes littoralis* under laboratory conditions. The fitness of the beetles at high RH was much better in all analyzed aspects: beetle survival, size and development time. Also, the thermogenesis at high RH was higher compared to low or medium RH, which had a synergistic effect on larval growth. The second factor, i.e. the parental effects, revealed a significant but rather minor effect in terms of development time and thermogenesis.

Other studies on the influence of RH on beetle development were mainly carried out on stored product pests e.g. larder beetles (Dermestidae: *Dermestes*). For *Dermestes maculatus* De Geer, it was noted that RH regulates the duration of larval stages, however, it does not influence the duration of the pupal stage^9^. The authors also observed that complete larval development occurred only at 70 and 100% RH. Similar patterns were confirmed for *Dermestes lardarius* L.^11^ and *Dermestes frischii* Kug.^10^. In the first study, the larval period was twice shorter comparing 80 and 40% RH. Moreover, the mortality was strikingly lower in these two humidity levels (5% in 80% RH and 65% in 40% RH). The mass of adult beetles also increased with RH, and it was doubled comparing extreme humidity levels. In the latter study, the two-fold differences were recorded, comparing the larval development, mortality and mass of adult beetles at 50% and 70% RH. The author emphasized that RH had an even more pronounced effect on larval development than temperature^10^. The elongation of development and an increase in mortality at low RH were also confirmed for other taxa^15,35,36^. Our findings align with these patterns. A pronounced influence of RH on larvae of beetles can be explained by the fact that they are largely vulnerable to desiccation due to their high activity (they constantly seek for food), thinner cuticle than in adult beetles, and rather elongated body. In the case of *Necrodes littoralis*, the larvae are strongly dependent on the food substrate that is digested externally. This process causes water loss^37^ and is likely affected by the environmental humidity. In the current study low RH resulted in drying out of the outer layers of meat, as a result of which, it became less accessible to the larvae. Under 50% RH it was extremely difficult for the larvae to effectively digest the meat. The difficulties in forming the feeding matrix on the upper surface of the meat forced the larvae to seek more favorable conditions, hence their activity and the formation of feeding cavities at the interface between the meat and the soil. Moreover, in the setups without adult beetles in the prelarval phase the matrix had to be formed by first instar larvae, which are relatively small, prone to desiccation and when there are few of them, they may not have been able to effectively form the matrix. The situation is slightly better when adult beetles create the matrix in the prelarval phase, then at low RH larvae can cope better with unfavorable conditions. When there are no adult beetles, the first instar larvae have very difficult task, especially under low RH, which was clearly demonstrated by the current results in 50% RH setup. Under low RH it is also very difficult to maintain a functional matrix, because it dries out constantly. On the other hand, in natural conditions larvae of *N. littoralis* feed in aggregations, hundreds of larvae can form such an aggregation, and this collective feeding enhances creation of the matrix on carrion. It has been shown that larval aggregations of *Necrodes littoralis* are beneficial in terms of mortality, development time and size of individual beetles, especially under unfavorable abiotic conditions^38^. Therefore, the larvae in our study could have performed better in the setup with low RH if we had tested larger aggregations.

The present findings that low RH is harmful for *N. littoralis* are in line with our previous research, which showed that these beetles are most abundant in humid habitats (e.g. alder forest) and seasons (i.e. spring)^24,34^. In the review of case studies from France, the authors referred to the Latin and vernacular names of these beetles (i.e. ‘littoralis’ and ‘shore sexton beetle’), which would suggest a preference for coastal areas. However, it was not supported later on, since *Necrodes littoralis* was found to be present mainly in cases located in forests, bushes and fields^22^. In Belgium, it was found mostly in agricultural sites, in spring and summer^39^. On the other hand, during our previous field studies, the most spectacular cases of cadaver colonization by these beetles were always recorded in an alder forest, in this case a forest formed around a small forest lake^24,34^. It is thus possible that these names reflect a preference for humid areas around inland water reservoirs. Although the aim of the current study was to find the optimal laboratory breeding conditions for *N. littoralis* beetles, we are aware that current findings cannot be fully transferred to natural death scenes. We used skinless meat and tested rather small larval aggregations, whereas under natural conditions these beetles colonize full cadavers and usually form massive larval aggregations^32^. Therefore, it is likely that the detrimental effects of low humidity can be attenuated under natural conditions by the cadaver quality and gregariousness of the larvae. However, still *N. littoralis* prefers cadavers in humid habitats, which reflects the importance of high humidity for this species.

We observed a higher larval mortality in the setup without adult beetles in 50% and 70% RH. A possible explanation could be that the feeding matrix was not present on meat surface when larvae started to feed. Larvae themselves form and expand the matrix^33^, but at low RH, when meat dries up quickly, this process might have been significantly impaired. In 90% RH, the differences in survival between colonies with and without adult beetles in the prelarval phase were smaller, indicating the importance of parental effects (i.e. formation of the matrix, pre-digestion of meat etc.) under less favorable environmental conditions. The preparation of the matrix by adult *Necrodes littoralis* beetles is important for the fitness and survival of juveniles^33^. Despite many advantages of the matrix, its formation is energetically costly, especially under conditions of reduced humidity. As terrestrial beetles are exposed to water loss by transpiration and excretion^37^, the latter may be of importance for *Necrodes littoralis*. To form the matrix these beetles spread oral and anal exudates over carrion^37^. It is probable though, that in low humidity the production of exudates is inhibited and the creation of the matrix is reduced.

Thermogenesis was generally low compared to our previous studies^31,33^. For *N. littoralis*, the heat production is casually related to the presence of a feeding matrix, therefore the most noticeable thermogenesis was observed in the highest RH setups. Moreover, the thermogenesis was more pronounced in the setup with adults’ presence in the prelarval phase, indicating that the activity of adult beetles in this phase and the resultant preparation of meat are important determinants of thermogenesis in the larval feeding phase and therefore the fitness of larvae.

In conclusion, high relative humidity increases survival rate, facilitates growth, shortens the development time for *N. littoralis* and promotes thermogenesis in the feeding matrix. It is therefore crucial to maintain high humidity conditions while rearing these beetles in the laboratory, both when they were sampled as insect evidence on a death scene and during experimental studies with this species.

### **Laboratory rearing protocol for*Necrodes littoralis***

**Fig. 6:**
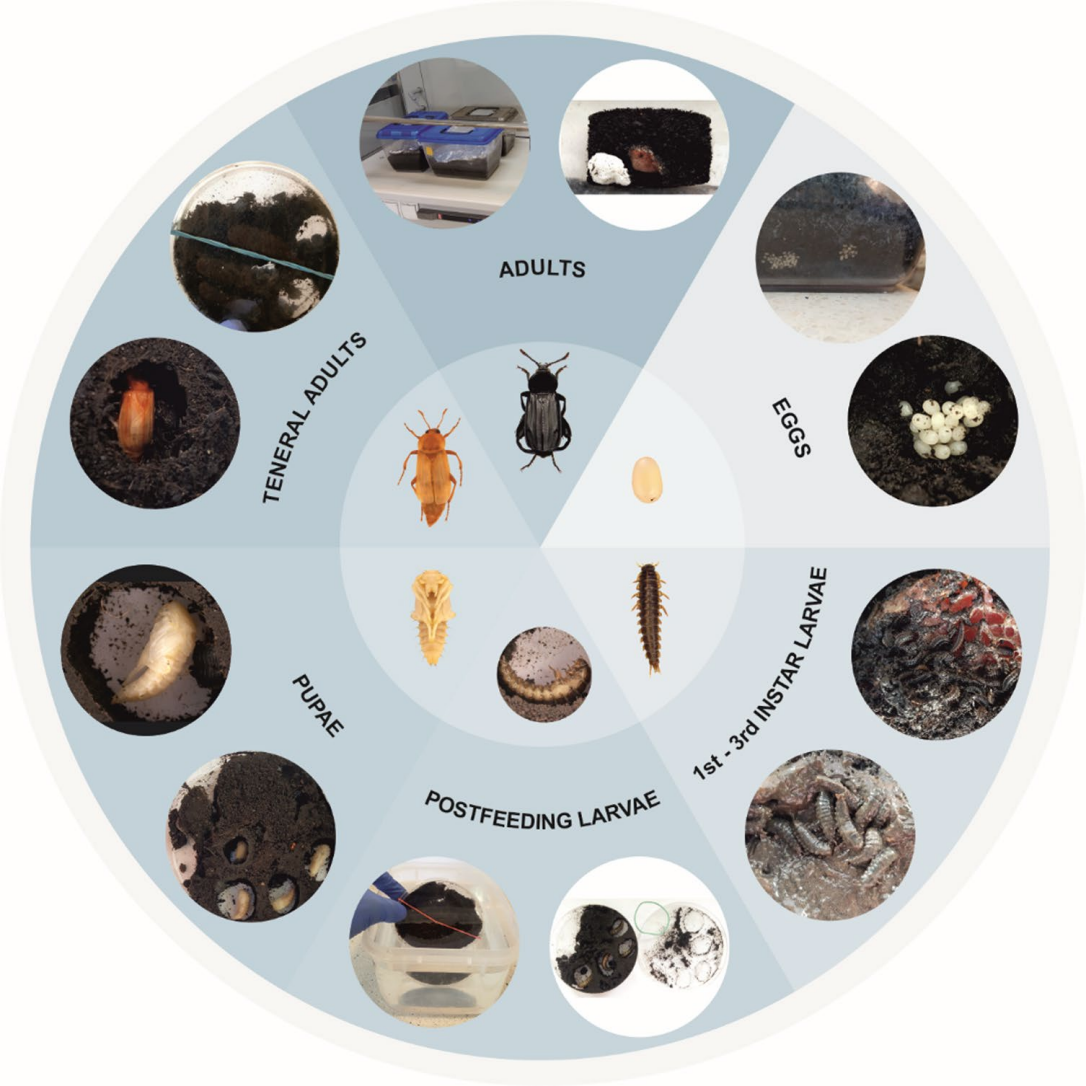
A simplified graphical rearing protocol for *Necrodes littoralis*.

Keeping in mind the lack of rearing protocols for insects important in forensic entomology and the significance of current findings about the RH influence on various aspects of the development of *Necrodes littoralis*, here we propose a comprehensive rearing protocol for this species (Table 3; Fig. 6). This protocol will be helpful when designing developmental experiments and, in particular, when rearing insect evidence from forensic cases. Further rearing of insect evidence can be beneficial mainly for gains in PMI accuracy. However, it may prolong the development and increase mortality of insects if not carried out properly^8^. In the current study, the difference in length of larval development between 50% and 90% RH at 23 °C was on average 5–6 days, which would have serious implications for post-mortem interval estimation. While developing the current protocol we tried to incorporate the guidelines on specific rearing requirements based on our experience and published data^30,32,33,38,40,41^. Consequently, when designing similar protocols for other forensically-important insects, we strongly encourage their authors to analyze several biotic and abiotic elements of a protocol, which are regularly important in terms of optimal rearing of insects e.g. collective or individual rearing^38,42^, cannibalism, diet^43,44^, photoperiod^45^, insect handling^46^ etc.

**Table 3:** A rearing protocol for *Necrodes littoralis*.

Developmental stage		Abundance	Container	Medium	Food	Abiotic conditions	Equipment	Others
**Imagines**	**Maintenance**	40–60	Transparent container (~ 7 l) with perforated lid	Soil (humid flower-growing substrate) to ^1^/_3_ height of container; cotton wool soaked with water, coverage (e.g. aluminum foil)	Pieces of raw pork meat *ad libitum*	Ambient light; 20–24 °C, humidity ≥ 90%	Fume hood, aluminum foil for humidity maintenance	Medium replacement once a week to avoid mite overgrowth
	**Reproduction**	10 (5♀+5♂)	Transparent container (~ 3.5 l) with perforated lid	Soil (humid flower-growing substrate) to ^1^/_2_ height of container; cotton wool soaked with water, coverage (e.g. aluminum foil)		16 L:8D; 20–24 °C, humidity ≥ 90%	Climate chamber or temperature chamber (in this case aluminum foil for humidity maintenance)	-
**Eggs**		several batches	Transparent container (~ 3.5 l ) with perforated lid		no	darkness; 20–24 °C; humidity ≥ 90%		Eggs should be covered with a layer of soil
**1st–3rd instar larvae**		> 10; at best 20–40	Transparent container (~ 7 l) with perforated lid	Soil (humid flower-growing substrate) to ^1^/_3_ height of container; cotton wool soaked with water, coverage (e.g. aluminum foil); additional insect mesh between container and lid	Pieces of raw pork meat *ad libitum*			Larvae can be kept with adults
**Postfeeding larvae**		4–6	Petri dish with ventilation (~ 90 mm diameter) closed with rubber gum	Soil (humid flower-growing substrate) soaked with water at the beginning; ~90% of dish volume	no	darkness; 20–24 °C; ambient humidity	Temperature chamber	Transfer to Petri dishes when larvae start to bury themselves; at 23 °C after 6–8 days from hatching
**Pupae**								-
**Teneral adults**								Keep teneral beetles in Petri dishes until they become fully colored. They may be cannibalized. Imagines lay viable eggs 7–14 days post eclosion

### Self-critique

The duration of the prelarval phase could have lasted longer. According to our previous research the thermogenesis was the highest when the prelarval phase lasted 5 days^33^. However, it was not possible due to the oviposition in experimental containers and the possibility of contamination of the colonies with additional larvae.

## Materials and methods

### Main colony

The laboratory colony was established using adult beetles collected in an alder forest of Biedrusko military range (52°31′N, 16°54′E; Western Poland) in 2017 (replenished with new samples in 2018 and 2022). The beetles were kept in plastic containers (7.5 l; 18 × 28 × 16.5 cm) with perforated lid, filled up to 1/3 with humid soil, with raw pork meat as food, cotton wool soaked with water to maintain water access and high RH and aluminum foil as a cover. Males and females were kept separately, 30–50 beetles per container. The colonies were supplied with food and water *ad libitum* and cleaned every 1–2 weeks to prevent excessive mite and mold growth.

### Experimental design

To test the influence of relative air humidity (RH) and parental effects on the development of *N. littoralis* we conducted the two-factor experiment. RH was analyzed on three levels: 50%, 70% and 90%, and parental effects on two levels: presence (parental effects: +) and absence (parental effects: –) of adult beetles in the prelarval phase. Each setup was replicated five times (five containers with adult beetles and five without in each RH level). The experiments were performed from November 2022 to February 2023, one humidity level was tested at a time (50% in November and December 2022, 70% in January and 90% in February 2023). All setups were kept at 23 °C and in darkness. Previous studies on the development of *Necrodes littoralis* showed that at this temperature, the mortality rate was the lowest^30^.

### Experimental procedures

**Fig. 7:**
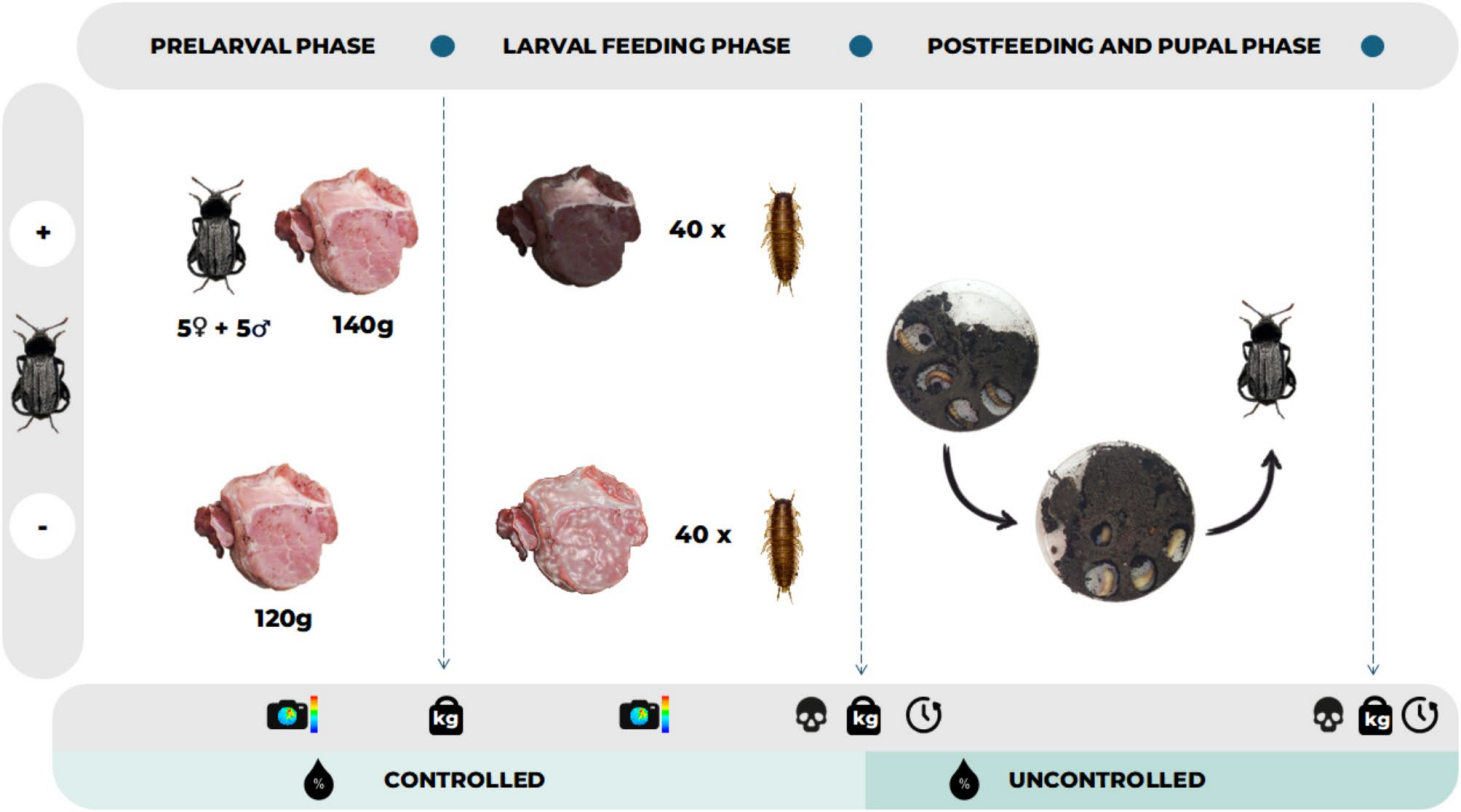
Graphical illustration of the experimental procedures. Symbols: ‘camera’ – thermal images, ‘weight’ – weighing, ‘skull’ – mortality; ‘clock’ – development time; ‘drop’ – humidity, ‘+‘- setup with adult beetles (parental effects +), ‘-‘ – setup without adult beetles (parental effects -).

For the experiments we used plastic containers (7.5 l; 18 × 28 × 16.5 cm), covered with mesh (instead of perforated lid), to ease the air circulation. The containers were filled up to 1/3 with soil. To maintain a given level of RH and temperature we used the climatic chamber (KK1200 SmartPro, POL-EKO, Poland). On day one, adult beetles (5 males and 5 females) in a ‘parental effects +’ setup were provided with 140 g of pork meat; whereas in a ‘parental effects –’ setup 120 g of meat was left without the beetles. The optimal amount of food was chosen based on previous results regarding the fitness of *N. littoralis*reared under various larval densities^31^. After four days, adult beetles were removed, meat was weighed and 40 first instar larvae per container were added in each setup. First instar larvae were chosen at random from the supplementary rearing containers established specifically for this purpose using adult beetles from our main colony. After reaching the postfeeding phase (larvae cease feeding and start to burrow into the soil), the larvae were counted, weighed (laboratory scale AS 82/220.R2, Radwag, Poland) and transferred to Petri dishes (90 × 14.2 mm, with ventilation, Noex) for further development in the temperature chambers with no control of RH (ST 1/1 BASIC or +, POL-EKO, Poland). Petri dishes were filled with soil, soaked with water and closed with rubber gum. Five larvae per dish were applied. At this point, we also weighed the leftover meat (kitchen scale Sencor SKS 4004YL). We monitored development landmarks: pupation and adult emergence. Freshly emerged adult beetles were weighed after they became fully colored and then were preserved in 70% ethanol for further measurements. Thermal images were taken daily during the prelarval and larval feeding phases using a thermal camera with 30° × 23° infrared lens (Testo 885-2; Testo, Germany) (Fig. 7).

### Data preparation and analysis

The independent variables (factors) were: ‘parental effects’ and ‘relative humidity’. The dependent variables were: colony mass at eclosion [g], average adult beetle mass [mg], total survival [%], average total, larval and pupal development times [days] size [number of specimens] and average thermogenesis in the prelarval and larval feeding phases [° C]. Results were analyzed using a 2-way analysis of variance (ANOVA) in Statistica 13 (TIBCO Software Inc., US). Fisher LSD test was used for post-hoc pairwise comparisons. Kaplan - Meier analysis was used to analyze survival in different setups. The sample size and subsamples were presented in the Supplementary material (Tables 2 and 3).

The colony mass at eclosion [g] was calculated by multiplying the number of eclosed adult beetles by their average (per colony) body mass. All eclosed adult beetles were weighed and their average body mass [mg] was used in the analyses. Survival was defined as the total number of adult beetles that successfully eclosed in the experiment, expressed in percentages of the initial colony size (40 larvae). Mortality was defined as the total number of premature beetles that died during the experiment.

The length of the elytra from the end of the scutellum, along the elytral suture, to the apical point of the elytra was measured, after drying a specimen with a paper towel and using Leica M165C stereomicroscope, with DFC450 camera and LAS software (Leica, Germany). The beetles are deposited at the Laboratory of Criminalistics (Adam Mickiewicz University, Poznań, Poland).

The following development phases (in days) were distinguished: larval development (from colony establishment to pupation), pupal development (from pupation to adult eclosion) and total development (from colony establishment to adult emergence).

Thermal images and temperature measurements were made with the emissivity set at 0.8 and the reflected temperature set at 17 °C. The average thermogenesis (during the prelarval or the larval feeding phases) was calculated by averaging daily thermogenesis quantified using thermal images of relevant colonies. This procedure included the following steps: (1) designating in the images the area of meat or the area covered by the feeding matrix; (2) averaging temperatures in these areas (75% of the pixels with the highest temperature were included to avoid the underestimation of the thermogenesis by cold spots from the cold soil particles); (3) selection of the background temperature: the average temperature of meat that was closest to 23 °C (usually the temperature in the 1st day upon establishment of a colony); (4) subtracting the background temperature from the meat/matrix temperature, which gave the daily thermogenesis. Thermal images were analyzed using R programming environment (version 4.3.0).

## Electronic supplementary material

Below is the link to the electronic supplementary material.

Supplementary Material 1

## Data Availability

The datasets used and/or analyzed in the current study are available from the corresponding author upon a reasonable request.
