# Supplementary material for: Guidelines for laboratory rearing of insect evidence: the importance of air humidity for breeding of Necrodes littoralis (L.) (Coleoptera: Staphylinidae)
Source: Sci Rep. 2025 Mar 12;15:8607. doi: 10.1038/s41598-025-92196-1 (PMC11903684; doi:10.1038/s41598-025-92196-1)
Supplement: Supplementary file 1 — Supplementary Material 1 [file 41598_2025_92196_MOESM1_ESM.docx]

**Supplementary Table 1 The results of the ANOVA test to assess the significance of ‘relative humidity’ and ‘parental effects’ on average length of elytra and total development time regarding sex of Necrodes littoralis beetles.**

| **Dependent variables** | **Sex** | **Independent variables** | | | | **Interaction** | |
| --- | --- | --- | --- | --- | --- | --- | --- |
|  |  | **Relative humidity** | | **Parental effects** | |  |  |
|  |  | F | *p* | F | *p* | F | *p* |
| **Average length of elytra** | f | 210.68 | <0.001 | 7.81 | <0.01 | 0.29 | 0.74 |
|  | m | 276.27 | <0.001 | 0.01 | 0.92 | 0.49 | 0.61 |
| **Average total development time** | f | 329.93 | <0.001 | 43.04 | <0.001 | 7.00 | <0.01 |
|  | m | 222.98 | <0.001 | 39.15 | <0.001 | 11.70 | <0.001 |

f – females, m – males.

**D**

**B**

**C**

**A**

**Supplementary Figure 1 The differences in the average length of elytra for females (A) and males (B), and total development time for females (C) and males (D).**


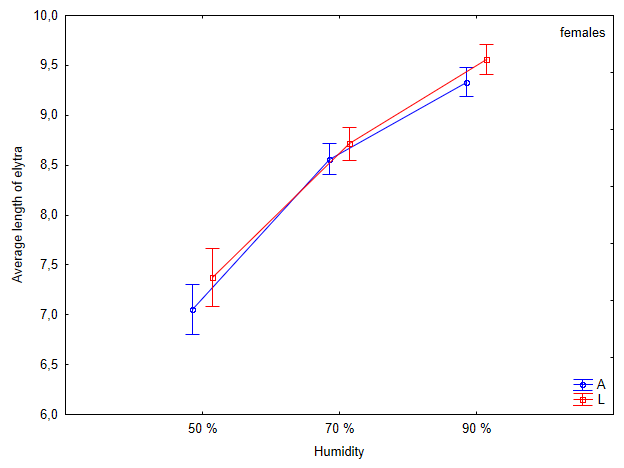

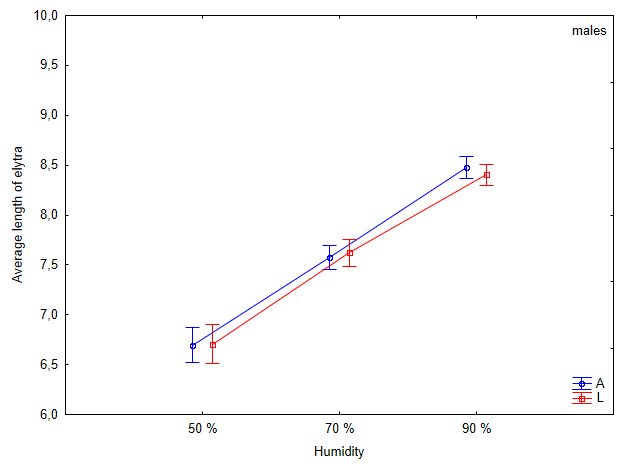

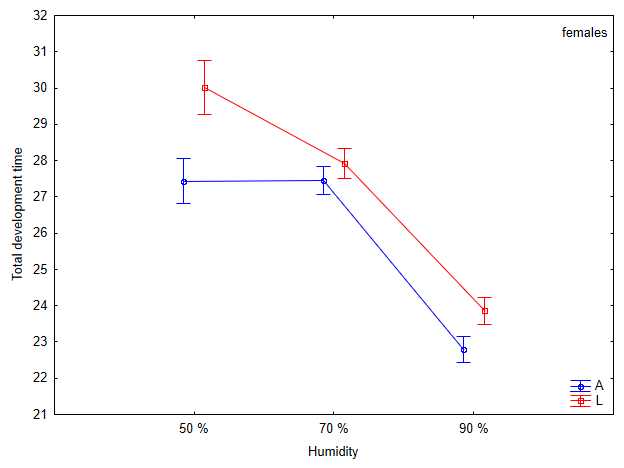

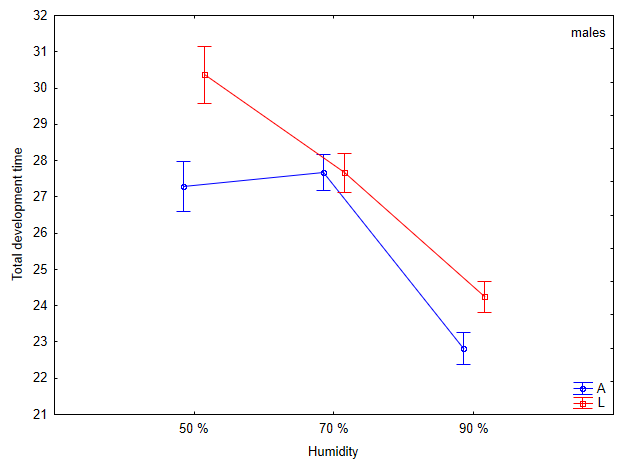


**Supplementary Table 2 Size and total development time of *Necrodes littoralis* beetles at eclosion according to experimental treatments and sex of the beetles.**

| **Humidity [%]** | **Setup** | **Sex** | **Number of specimens** | **Average length of elytra [mm]** | | **Total development time [days]** | |
| --- | --- | --- | --- | --- | --- | --- | --- |
|  |  |  |  | *m* | *r* | *m* | *r* |
| **50** | A | f | 29 | 7,05 | 4,94 - 8,29 | 27,43 | 26 - 31,08 |
|  |  | m | 35 | 6,69 | 5,58 - 8,13 | 27,29 | 25 - 32 |
|  | L | f | 20 | 7,37 | 6,45 - 8,30 | 30,02 | 27 - 35,25 |
|  |  | m | 27 | 6,70 | 5,31 - 7,64 | 30,37 | 26,25 - 35,25 |
| **70** | A | f | 70 | 8,56 | 6,34 - 9,92 | 27,45 | 24,25 - 31,95 |
|  |  | m | 70 | 7,58 | 5,59 – 8,81 | 27,67 | 24,25 - 45 |
|  | L | f | 65 | 8,71 | 6,63 - 9,88 | 27,91 | 25,95 - 34,95 |
|  |  | m | 57 | 7,62 | 6,40 - 8,40 | 27,67 | 25,25 - 35,96 |
| **90** | A | f | 86 | 9,33 | 5,78 - 10,46 | 22,79 | 20,91 - 27,92 |
|  |  | m | 90 | 8,48 | 7,36 - 9,56 | 22,82 | 20-92 - 27,92 |
|  | L | f | 78 | 9,56 | 8,45 - 10,54 | 23,86 | 21,17 - 32,92 |
|  |  | m | 93 | 8,40 | 7,51 - 9,37 | 24,24 | 22,08 - 31,92 |

A - adults present in the prelarval phase, L – adults absent in the prelarval phase; f – females, m – males; *m* – mean. *r* - range.

**Supplementary Table 3 Size of the subsamples used in the experiments.**

|  |  |  | **Treatments** | | | | | | | | | | | | |
| --- | --- | --- | --- | --- | --- | --- | --- | --- | --- | --- | --- | --- | --- | --- | --- |
| No. | **Dependent variables** | **N** | **50% RH** | | | | **70% RH** | | | | **90% RH** | | | | |
|  |  |  | A | | L | | A | | L | | A | | L | | |
|  |  |  | *m* | *r* | *m* | *r* | *m* | *r* | *m* | *r* | *m* | *r* | *m* | *r* |  |
| 1. | **Av. adult beetle mass** | 5 | 12,8 | 4 - 25 | 9,4 | 3 - 26 | 28 | 17 - 36 | 24,4 | 20 - 30 | 35 | 30 - 40 | 34,2 | 23 - 38 |  |
| 2. | **Total survival** | 5 | 12,8 | 4 - 25 | 9,4 | 3 - 26 | 28 | 17 - 36 | 24,4 | 20 - 30 | 35 | 30 - 40 | 34,2 | 23 - 38 |  |
| 3. | **Av. total development time** | 5 | 12,8 | 4 - 25 | 9,4 | 3 - 26 | 28 | 17 - 36 | 24,4 | 20 - 30 | 35 | 30 - 40 | 34,2 | 23 - 38 |  |
| 4. | **Av. larval developmental time** | 5 | 28,8 | 24 - 32 | 21 | 12 - 32 | 34,8 | 32 - 38 | 29,6 | 26 - 34 | 36 | 32 - 40 | 35,6 | 24 - 40 |  |
| 5. | **Av. pupal development time** | 5 | 28 | 24 - 31 | 20,4 | 12 - 32 | 32 | 26 - 37 | 28,6 | 25 - 32 | 35,8 | 32 - 40 | 35,4 | 23 - 40 |  |
| 6. | **Av. thermogenesis in the prelarval phase** | 5 | 5 | - | 5 | - | 5 | - | 5 | - | 5 | - | 5 | - |  |
| 7. | **Av. thermogenesis in the larval phase** | 5 | 9,6 | 9 - 10 | 11,4 | 10 - 12 | 10,2 | 9 - 11 | 10,8 | 10 - 12 | 8 | - | 9,2 | 8 - 10 |  |

N - number of replications for each experimental setup, A - adults present in the prelarval phase, L – adults absent in the prelarval phase; *m* – mean. *r* - range. Subsample – number of beetles (No. 1 - 5) or number of thermal pictures taken on a daily basis ( No. 6 - 7)
